# Effects of seasonal meteorological variables on *E. coli* persistence in livestock faeces and implications for environmental and human health

**DOI:** 10.1038/srep37101

**Published:** 2016-11-15

**Authors:** David M. Oliver, Trevor Page

**Affiliations:** 1 Biological & Environmental Sciences, University of Stirling, Stirling, FK9 4LA, UK; 2 Lancaster Environment Centre, Lancaster University, Lancaster LA1 4YQ, UK

## Abstract

Agriculture contributes significant volumes of livestock faeces to land. Understanding how faecal microbes respond to shifts in meteorological patterns of contrasting seasons is important in order to gauge how environmental (and human health) risks may alter under a changing climate. The aim of this study was to: (i) quantify the temporal pattern of *E. coli* growth within dairy faeces post defecation; and (ii) derive *E. coli* seasonal population change profiles associated with contrasting environmental drivers. Evaluation of the die-off dynamics of *E. coli* revealed that a treatment mimicking drought and warming conditions significantly enhanced persistence relative to *E. coli* in faeces that were exposed to field conditions, and that this pattern was consistent across consecutive years. The internal temperature of faeces was important in driving the rate of change in the *E. coli* population in the immediate period post defecation, with most *E. coli* activity (as either die-off or growth) occurring at low dry matter content. This study highlighted that the use of seasonal *E. coli* persistence profiles should be approached with caution when modelling environmental and human health risks given the increased likelihood of atypical seasonal meteorological variables impacting on *E. coli* growth and die-off.

The sustainability of land and water resources is under pressure due to growing demands on agricultural systems to deliver food for an increasing world population^1^. One source of pressure can be attributed to livestock agriculture and the production of large volumes of faeces and associated manures^2^. If managed effectively, faecal contributions to agricultural land from livestock can serve as a valuable farm resource and organic fertiliser, but if mismanaged and combined with opportunities for hydrological transfer, they can threaten water quality draining from catchment systems with a range of pollutants.

Microbial pollution of water from livestock faeces is a particular concern given the potential for transmission of zoonotic disease agents such as *E. coli* O157, *Campylobacter jejuni* and *Cryptosporidium parvum*^3^,^4^. The quantification of faecal indicator organisms (FIOs), such as *E. coli*, is used routinely around the world to infer the hygienic status of water resources. Whether or not pathogenic microorganisms are actually present when FIOs are detected in water is difficult to determine without further analysis, and FIOs do not necessarily equate to the presence of specific waterborne pathogens^5^. However, the presence of FIOs in the aquatic environment does indicate contamination with material of faecal origin, with higher concentrations of FIOs suggesting increased risk to human health with respect to minor, self-limiting illness (principally gastroenteritis)^6^.

Climate change represents a significant emerging threat to human health on Earth^7^. For example, waterborne illness, currently responsible for over 3.4 million deaths every year, is expected to increase because of predicted climate-driven impacts on water quantity and quality^8^. Certainly, the regulation of microbial water quality standards, which is linked to culturable counts of FIOs, will be challenged due to the likelihood of increasing frequency of storms under a changing climate. Storms, and resulting high energy flows of runoff, often result in elevated FIO counts in receiving waters because of more frequent spills from sewage overflows and contributions from diffuse sources in agricultural catchments^9^.

Sewage overflows can be targeted with investment from water utilities and significant improvements have been made in recent years to protect water quality (and human health) from reduced microbial loads contributed from combined sewer overflows, e.g. via increased storage and sewerage infrastructure^10^. Managing catchment sources of FIOs in the face of climate change presents a greater challenge because of the diffuse nature of the loading of agricultural slurries, manures and faeces, which are applied or deposited across large areas of farmed landscapes. Furthermore, livestock excretions direct to pasture undergo no treatment process and, therefore, the microbial load of faeces is often high. Thus, livestock faecal deposits present an environmental and human health risk if, for example, FIOs and potential pathogens are mobilised from the faecal matrix following rainfall events and are subsequently transferred into the wider catchment system, increasing opportunities for human exposure^11^.

Understanding the sensitivity of FIO persistence to shifts in rainfall and temperature patterns across different seasons is therefore important in order to gauge how environmental (and human health) risks may alter under a changing climate. This is especially true given reported occurrences of FIO regrowth in livestock faeces under conducive environmental conditions^12^,^13^,^14^,^15^. Whether climate change impacts are extreme or subtle, many of the most interesting, challenging and underexplored research questions concerning FIO responses to climatic shifts in catchment systems are therefore associated with their behaviour in faecal sources, their capacity to increase in number when conditions allow and the implications of such FIO population growth for environmental regulation of public health significance^16^. The aim of this study was to: (i) undertake targeted quantification of the temporal pattern of FIO population dynamics within dairy faeces post defecation and determine whether the occurrence of FIO population growth exhibits a consistent seasonal trend across consecutive years; (ii) derive much needed seasonal population change profiles associated with contrasting environmental drivers such as episodic rehydration and temperature shifts; and (iii) use these data to explore environmental and human health risk implications of seasonal meteorological variables impacting on *E. coli* population dynamics in livestock faeces.

## Results

**Figure 1:**
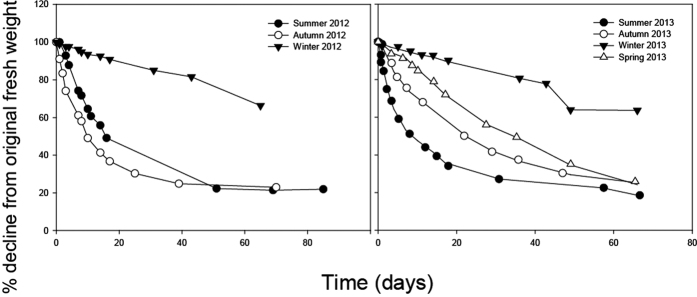
Mean percentage decline in cowpat fresh weight over time for different seasons of 2012 and 2013 (note spring data are missing from 2012 sampling campaign).

**Figure 2:**
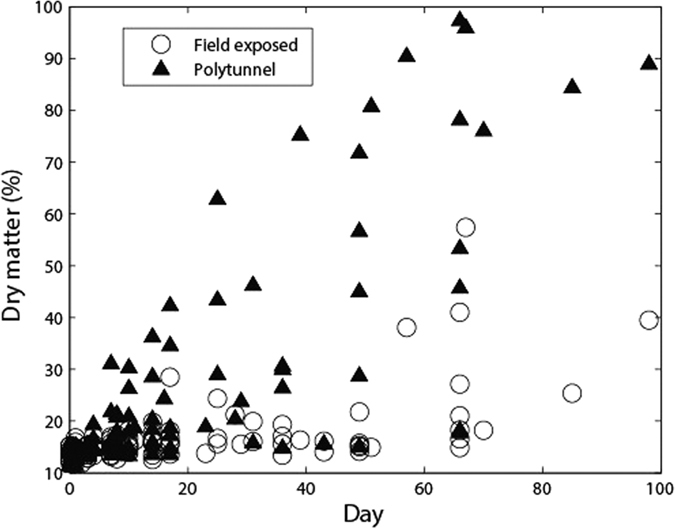
Relationship between percentage dry matter (DM) content of dairy cowpats and their age (d) since excretion.

All culture method blanks were negative for *E. coli* indicating that no cross contamination occurred during sample processing. Changes in the moisture content over time, represented as a percentage decline from the original fresh weight of dairy faeces, kept under the polytunnel treatment are shown in Fig. 1. Summer and autumn demonstrated a more rapid decline in cowpat mass over time relative to winter (in both 2012 and 2013) and spring (2013 only; 2012 = nd). Comparative data for the field exposed treatments are unavailable but Fig. 2 highlights the change in percentage dry matter with increasing time for both treatments and indicates that ~3 days post defecation (equivalent to ~17% dry matter content, irrespective of treatment) is the point at which the polytunnel and field exposed cowpats start to diverge with regard to their dry matter characteristics. The highest recording of dry matter for field exposed cowpats was 58% whereas cowpats exposed to the polytunnel treatment recorded up to 98% dry matter.

**Figure 3:**
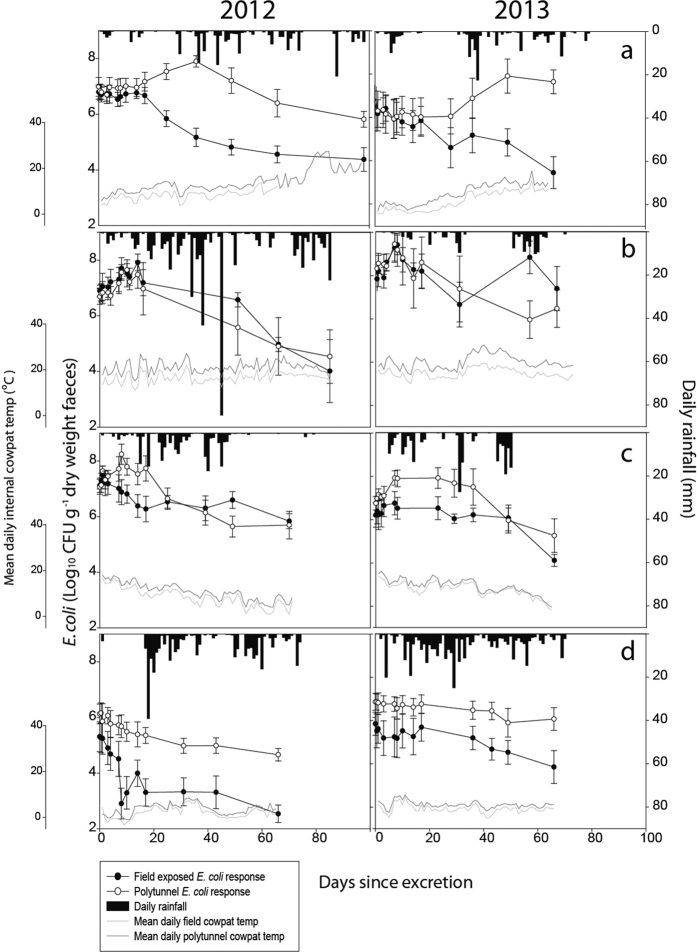
*E. coli* population numbers over time for spring, summer, autumn and winter (**a**–**d**, respectively) in 2012 and 2013. Data points are the mean of five replicates ± the standard error. Rainfall only applies to the field exposed treatment.

Changes in *E. coli* concentrations over time under different treatments of open field exposure or polytunnel protection, and across all seasons of two consecutive years are shown in Fig. 3. Overall, no significant difference in *E. coli* counts were recorded between 2012 and 2013 (P > 0.05). Daily rainfall distribution over each of the sampling periods was recorded for the field exposed cowpats to provide environmental context alongside the daily average internal temperature of the cowpat environment (Fig. 3). The weather characteristics associated with each season during the two years of this study accommodated some interesting patterns and extremes in meteorological records for the UK, and a summary of the seasonal characteristics associated with rainfall, air temperature and internal cowpat temperatures is provided in Table 1.

Evaluation of the die-off dynamics of *E. coli* revealed that the polytunnel environment significantly enhanced *E. coli* persistence in dairy faeces relative to cowpats that were field exposed, and that this pattern was consistent across years (P < 0.001). Season was also found to significantly impact *E. coli* persistence (P < 0.001). In 2012 both summer and autumn promoted higher *E. coli* counts relative to spring and winter for all treatments combined, whereas in 2013 summer alone accommodated the highest *E. coli* counts (P < 0.001). Spring 2013 represented the only period of study whereby counts of *E. coli* were found to be significantly higher at the end of the experiment relative to day 0 (P < 0.001), though this was only true for cowpats held under the polytunnel treatment (0.71 log_10_ CFU g^−1^ dry weight faeces higher than day 0 on day 66). A significant interaction between treatment and season was recorded for both years of the study. Fitted means for both the field exposed and polytunnel treatments of the summer experiments aligned closely, but spring, autumn and winter all accommodated persistence profiles for the polytunnel *E. coli* populations that resulted in a deviation of counts from those observed in the field exposed cowpats (P < 0.001).

Evidence of *E. coli* growth post-defecation was observed for both treatments and across multiple seasons. *E. coli* populations within dairy faeces exposed to the polytunnel treatment exhibited growth in spring, summer and autumn for both 2012 and 2013. Growth in *E. coli* populations associated with the field exposed cowpats was only recorded in summer, and this was consistent for both 2012 and 2013. In the majority of cases the growth period occurred in the first 10 days post-defecation; however, a number of notable exceptions were observed. For the polytunnel exposed cowpats, the *E. coli* populations associated with the spring faeces were slower to demonstrate a population increase. For example, in 2012 the peak *E. coli* concentration (7.90 log_10_ CFU g^−1^ dry weight faeces; representing 0.92 log_10_ growth) occurred much later, on day 36, and in 2013 the peak population (7.40 log_10_ CFU g^−1^ dry weight faeces; also representing 0.92 log_10_ growth) was recorded on day 49. The period of growth appeared to be delayed following a stationary phase in the *E. coli* population. The other delayed growth phase that registered in the later stages of post defecation was associated with the field exposed cowpats during the summer of 2013, with cell numbers increasing from 6.38 to 8.08 log_10_ CFU g^−1^ dry weight faeces (increase of 1.7 log_10_ CFU) between days 31 and 57 during a second phase of regrowth. Details of the more typical growth phases in the immediate period post defecation are reported in Table 2 with the field exposed cowpats in summer 2013 registering the largest increase in *E. coli* concentrations for an initial period of growth (1.19 log_10_ CFU g^−1^ dry weight faeces), which was approximately 0.5 log_10_ CFU g^−1^ dry weight faeces less that the secondary phase of growth recorded in cowpats held under this treatment approximately 49 days later. The most rapid rate of *E. coli* decline was observed during winter 2012 within the field exposed cowpats. The population experienced a 2 log_10_ reduction in CFU over ~14 days in the immediate period post defecation, and after 66 days concentrations were close to limits of detection having reached a level of 2.54 log_10_ CFU g^−1^ dry weight faeces.

**Figure 4:**
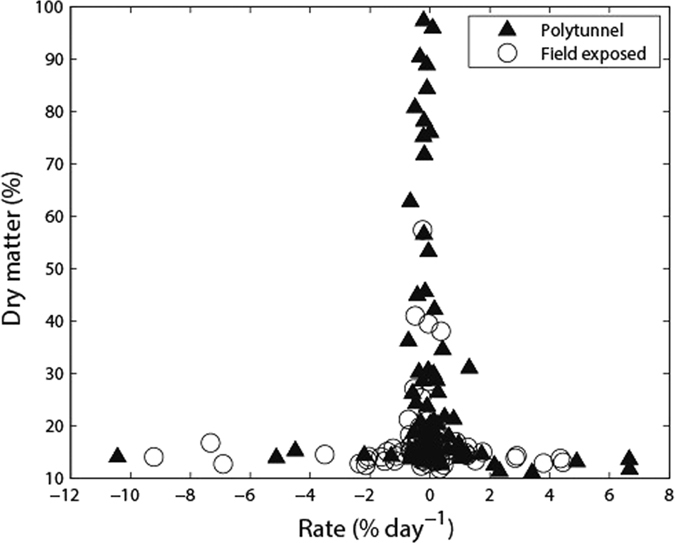
Relationship between rate of change in *E. coli* concentrations and % dry matter content. Note that for the rate of change, 100% represents the previous observed value, i.e.% change from previous value but expressed as a per day value for consistency.

**Figure 5:**
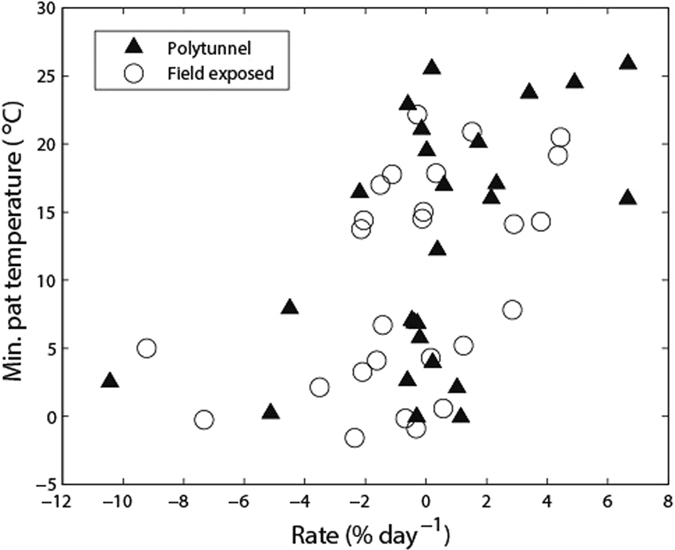
The rate of change in *E. coli* population in the faecal matrix as a function of minimum cowpat temperature in first 3 days post excretion. Note that for the rate of change, 100% represents the previous observed value, i.e.% change from previous value but expressed as a per day value for consistency.

The rate of change in *E. coli* population in the faecal matrix as function of dry matter content, and as function of minimum cowpat temperature in first 3 days post excretion is shown in Figs 4 and 5, respectively. From Fig. 4 it can be seen that all high rates of *E. coli* population change (both net die-off & net growth) occur at low dry matter content. As the cowpats dried out there was a tendency for only die-off. Figure 5 combines data from both field exposed and polytunnel treatments for the first three days post-defecation to show the rate of change in cell numbers versus minimum cowpat temperature. The distinction between polytunnel and field exposed cowpats was not considered important given the spread of the values for both treatments. Minimum cowpat temperature was chosen as an explanatory variable because it can represent an important factor in cell die-off; the relationship reported in Fig. 5 was similar for both mean and maximum cowpat temperature.

## Discussion

This study has provided important field relevant data on *E. coli* persistence patterns in dairy faeces over two consecutive years in the UK. A targeted high resolution sampling strategy was used to profile, in detail, how culturable *E. coli* populations in freshly excreted dairy faeces responded to contrasting environmental conditions. While a number of studies have reported on *E. coli* regrowth in animal faeces under field relevant conditions e.g. refs 17, 18, 19, few, if any, have explored this growth phase across multiple seasons of *consecutive years*, and compared *E. coli* growth response across both field exposed cowpats and those held under an elevated drought/temperature treatment. Understanding the behavior of microbial pathogens and faecal indicators in the environment is essential in helping to minimise human health risks linked to contact with unclean water^20^. This study therefore enabled an investigation into how sensitivities in meteorological variables might impact on the response of *E. coli* populations in a common faecal source found in agricultural systems, and whether the definition of ‘seasonal’ die-off rates for *E. coli* are appropriate.

Changes in the *E. coli* numbers observed throughout the study were a combination of cell growth, die-off and, for the field exposed cowpats, dispersal following rainfall. The repeat of all four seasonal experiments over a 24-month study period proved advantageous. It enabled capture of a range of contrasting environmental conditions that can occur for any given season in the UK and highlighted year-on-year variability in weather patterns for the same season. The period of study also enabled an opportunistic investigation of some uncharacteristic weather extremes through different seasons. For example, spring 2012 included an unseasonably warm week-long period followed immediately by freezing conditions (representing a drop in temperature of 27 °C from one week to the next). Summer 2012 was one of the wettest years on record in eastern Scotland and spring 2013 was extremely cold and wintery, and one of the coldest on record for the UK^21^. Finally, winter 2013/14 was, at the time, the wettest winter since records began in the UK^22^. While atypical, such patterns are likely to become more common in the future with data from the IPCC and global climate models suggesting that a future world climate will experience higher temperatures, more frequent and extreme heat waves and more regular patterns of intense rainfall^23^.

Research has explored potential future scenarios of land management and climate change on microbial water quality and reported that climatic factors such as trends in seasonal and annual precipitation will deliver the most significant impact on microbial fate and transport in catchment systems^24^. The uncharacteristic extremes in weather variables recorded during our study were valuable in driving periods of activity in *E. coli*, and thus help to develop our understanding of temperature and moisture effects on *E. coli* population behaviour in livestock faeces. For example, a mild temperature spell in mid-April 2013 (~day 30 onwards, Fig. 3) was coincident with a defined period of *E. coli* growth in cowpats held in the polytunnel environment. Internal cowpat temperatures in cowpats reached a peak temperature 37.5 °C during that milder period, reflecting near perfect conditions of 37 °C needed to promote the most efficient *E. coli* replication^25^. Other notable changes in *E. coli* populations with weather variables were observed in summer 2013, this time for the field exposed cowpats. The resurgence in cell numbers between days 31 and 57 appeared to coincide with a period of rainfall, which would have rehydrated the faecal matrix and likely led to the redistribution of nutrients, thus benefitting the survival of *E. coli* via moisture delivery and nutrient provision^14^,^18^,^26^. In addition, Table 1 shows that the field exposed summer 2013 cowpats also registered peak hourly internal temperatures of 39.5 °C, again very close to the ideal temperature to promote *E. coli* multiplication which, when coupled with the rehydration from rainfall, looks to have created optimal conditions for an increase in *E. coli* numbers despite the faeces being over 57 days old. Interestingly, the field exposed cowpats in summer 2012 also warmed to a similar maximum hourly temperature (38.9 °C, Table 1) but on this occasion no *E. coli* growth was observed as the cowpats aged beyond 20 days. The notable difference recorded during summer 2012 that might help explain the lack of observed regrowth was the amount (and patterns of distribution) of daily rainfall. This was far higher than that experienced in 2013 and most likely led to the detachment of organic material and associated cells from the faecal pats through processes of raindrop impact and subsequent mobilisation and wash-out of *E. coli* from the faeces into the surrounding environment^11^,^27^.

It is important to note that the polytunnel environment did promote very high temperatures, and these clearly exceeded what we would expect in the UK, but they provide useful context for transferring our findings to areas of the world where higher summer temperatures are common. Of particular interest was the possible detrimental effect of the internal cowpat temperature when the hourly maximum exceeded 43 °C. In the polytunnel treatment the maximum hourly cowpat temperature was logged as 43, 46.5 and 50.5 °C for spring and summer 2012 and summer 2013, respectively, during which cell decline was observed. It is not possible to say with certainty that those temperatures alone were responsible for driving the decline in culturable *E. coli* counts, because indirect effects of dehydration in the faecal matrix will also play a role. However, these findings are complementary to earlier work on *E. coli* O157 which suggested that prolific growth of this particular strain was observed when cultures were held between temperatures of 30 and 42 °C, with cell growth becoming less effective above this temperature range^28^.

The time taken to reach peak regrowth of *E. coli* (highest value of *E. coli* that was recorded subsequent to values determined upon excretion) in our study was generally between one and two weeks, apart from the exceptions already discussed. Others who have studied the regrowth phase of *E. coli* in dairy cowpats across different seasons in Virginia, USA recorded peak regrowth after 7, 7 and 4 days for spring, summer and autumn, respectively^13^. It can, however, be difficult to identify the exact timing of peak regrowth depending on the sampling frequency used. In our study we adopted a high frequency sampling regime to profile the regrowth phase, collecting nine samples in the first two weeks. By contrast, Soupir *et al.*^13^ sampled dairy cowpats every two to three days for the first seven to ten days before reverting to a weekly sampling schedule and so clearly the recorded day of peak *E. coli* is dependent, to some extent, on the sampling days. The maximum increase of cell numbers during a regrowth phase in the current study (1.7 log_10_ CFU g^−1^ dry weight faeces, summer 2013, field exposed) aligned closely with the 1.5 order of magnitude increase observed by Sinton *et al.*^14^ during their investigation of *E. coli* persistence in bovine faeces across different seasons in New Zealand though, as mentioned earlier, our largest observed regrowth was a secondary growth phase later in the seasonal monitoring period, and not immediately post defecation.

Impacts of the variable meteorological conditions over the course of the repeated seasonal experiments have highlighted that a degree of caution is needed when relating ‘seasons’ as indicators of FIO die-off rates. Instead, it is the climatic/meteorological variables that should take priority because, for any given year, there might be an atypical season, e.g. a particularly hot spring or a very wet summer. Some generic rules can of course be identified – for example, winter does appear to be consistent in generating dairy faeces with substantially lower *E. coli* counts relative to other seasons. However, that observation applies to *E. coli* shedding and not persistence, and the milder winter of 2013 appears to have benefitted the *E. coli* population based on the subsequent persistence profiles observed in the field exposed cowpats in 2013 relative to 2012, thus reinforcing the nuances of within-season variability in *E. coli* persistence profiles. The use of the Q_10_ model by Martinez *et al.*^18^ to simulate *E. coli* survival in bovine faeces with respect to temperature, drawing on datasets from around the world, highlighted a relationship between the average temperature recorded during the first week after faecal deposition and the growth rate per unit of thermal time. However, that research also highlighted the need to better understand the factors that define the specific characteristics of the growth phase (e.g. magnitude and duration of growth) prior to net die-off of cells.

The divergence of dry matter content, after day 3, in faeces held under different treatments provided an objective way of setting a threshold (17% dry matter content) with which to explore temperature effects on *E. coli* population response in dairy faeces. Our assumption was that after day 3 we may start to observe drying or nutrient limitation effects on *E. coli* population responses rather than strict temperature impacts, and Sinton *et al.*^14^ noted that water content was a key factor in defining the growth profile of indicator bacteria in bovine faeces. Our regression analysis revealed that there was a real but scattered relationship between the likelihood of regrowth or die-off in the initial period after deposition and the minimum cowpat temperature (which can be related to air temperature) and that most *E. coli* activity is occurring at a dry matter content of less than 20%. Above this dry matter threshold it is likely that the rate of change of *E. coli* populations (positive or negative) is slow, unless rainfall rehydrates the faecal matrix (e.g. as experienced by the summer field exposed cowpats in 2013). Interestingly this relationship is weakened with increasing dry matter content suggesting the relationship observed in Fig. 5 begins to change or break down with faecal dehydration, thus highlighting the complex interaction between moisture content and internal temperatures of dairy faeces in determining the resulting *E. coli* persistence patterns^29^. This type of statistical relationship can prove useful in adding more robust and nuanced detail to models that attempt to predict the accumulation of FIOs on land (e.g. ref. 30).

The diet of the cows was necessarily different during contrasting seasons of study, reflecting the grazing and housed periods. During grazing, which was typical between April through October, the cattle diet was predominantly perennial ryegrass *Lolium perenne*, supplemented with dairy cake (an 18% protein mix containing wheat and distiller’s grains) during milking. When housed, the cattle diet consisted mainly of silage combined with distiller’s grains, brewer’s barley and molasses, which was supplemented with dairy cake at an increased 20% protein mix during milking. The lower counts of culturable *E. coli* that were observed at excretion during winter sampling campaigns may therefore relate to predominant intake of the silage diet during this time. However, the lower counts in winter may also be a result of reduced opportunity for continuous ingestion of *E. coli* given that the cows are no longer grazing on pasture that is undoubtedly contaminated with *E. coli* from their own faeces^31^,^32^. It was not the intention of our study to monitor coprophagous invertebrate and fungi on cowpats though qualitative observation of their colonisation of cowpats over time was noted. Future investigations to consider how such colonisation varies between treatments, and the potential impacts of such colonisation on study populations would be an interesting area of future research. There is, therefore, clear rationale for a growing portfolio of high resolution studies of *E. coli* persistence under field relevant conditions, so that the research community can build stronger relationships between *E. coli* behaviour in the environment and complex interacting meteorological and ecological controls.

Our results have important implications for those interested in understanding wider environmental risks, e.g. contamination of recreational waters such as rivers or bathing waters with FIOs. Knowledge of the drivers of surface water contamination with microbial pollutants can be reinforced by more detailed understanding of potential sources and their risks^33^ and by using catchment scale modelling^16^. Indeed, different modelling approaches can be used, often combining hydrological information with livestock grazing densities and associated *E. coli* content of faecal matter to inform regulators and decision-makers of spatial and temporal risks in catchment systems^34^. However, this can be a challenging undertaking due to the naturally high variation of *E. coli* in different environmental matrices in both space and time^35^,^36^, further reinforced by our study and the complex patterns observed in *E. coli* persistence (both in terms of growth and die-off) across multiple seasons and years.

Do growth and regrowth dynamics of *E. coli* in the environment challenge the indicator paradigm in a changing world? FIOs detected in environmental samples, e.g. regulated water bodies, provide evidence that a faecal source has, in some way, connected with an aquatic receptor^37^. Whether or not the faecal pollution event was recent is more difficult to determine, especially with mounting evidence that temperature drivers (as seen with the polytunnel treatment and summer experiments) and rehydration can not only prolong the persistence of *E. coli* in faecal sources in agricultural landscapes but also promote repeated growth phases. Nonetheless, the presence of FIOs, such as *E. coli*, in waterbodies where large numbers of people are likely to be exposed, suggests increased likelihood of human contact with microorganisms associated with material of faecal origin, irrespective of whether or not *E. coli* has undergone a period of growth or regrowth during its transition from a faecal source to an aquatic receptor. Provided that we can guard against the common misconception that high FIO presence is always indicative of pathogen presence, then the FIO paradigm remains useful as a water quality standard to not only inform on the level of general faecal pollution in the environment but also underpin ‘health-based’ criteria for water exposure.

Temperature and dry matter content of faecal material are important factors for controlling the response of *E. coli* persistence patterns in aging dairy faeces. However, uncertainty over exact thresholds of temperature-moisture-rainfall induced (dis)benefits on *E. coli* persistence adds to the challenge of sustainable water resource protection from diffuse microbial pollution. Undoubtedly, ‘seasons’ can be variable and they will not always represent a useful framework for assigning FIO die-off rates in models that attempt to predict FIO accumulation on land. A more sophisticated handling of the influence of climatic variables is likely to be more useful in this respect and field relevant experiments that have profiled *E. coli* persistence in livestock faeces have allowed us to move away from the simplistic laboratory-based assumption of microbial first-order decay following excretion from the livestock gut. While meteorological factors such as air temperature and rainfall will influence the persistence profiles of *E. coli* in livestock faeces, it is worth recognising that those same meteorological factors will also impact on how land is managed. Such larger scale decision-making may potentially over-ride some of the more subtle details of *E. coli* dynamics associated with the scale of an individual cowpat. Understanding how this complex mix of environmental, biological and management-related drivers contribute to *E. coli* source loading across agricultural landscapes remains a key focus for catchment and public health protection.

## Materials and Methods

### Sample collection

Twelve fresh dairy cowpats were collected from a conventional 165 ha dairy farm in Stirlingshire, Scotland, on eight sampling occasions over a two year study spanning 2012–2013. This resulted in the collection of 96 cowpats in total. Cowpats were collected at the beginning of March, June, September and December of each year and represented faeces excreted at the start of spring, summer, autumn and winter in the northern hemisphere, respectively. The twelve cowpats served as replicates and were collected from twelve different cows on each sampling occasion, all collected within 30 minutes of excretion. The dairy herd comprised 80 head of Holstein Friesian cattle, with cows normally housed from October through to the end of March, and pasture grazing typically from April to September. Fresh cowpats were transferred to clean circular plastic saucers (12 inch diameter) using a sterilised spade (70% Industrial Methylated Spirit (IMS), rinsed with sterile water) and were collected from a covered holding-barn that was used during the transfer of dairy cows to the parlour for morning milking. A mechanical scraping system cleaned the barn floor twice daily and so all cowpats collected were assured to be fresh deposits.

Our experiment aimed to evaluate the role of temperature and moisture content of faeces on *E. coli* populations under field conditions. Ten of the twelve cowpats collected at the start of each seasonal experiment were sampled and analysed for *Escherichia coli* and dry matter content during each seasonal experiment. The other two cowpats served as indicators of internal cowpat temperature and had a DS1921G Thermochron i-button temperature logger (iButtonLink, WI, USA) placed within the core of the faecal matrix. The ten cowpats were randomly divided into two treatments (n = 5) representing field-exposed and polytunnel protected cowpats and transferred to these treatment locations within 20 minutes of collection from the dairy farm. Cowpats were transported from the farm in individual circular saucers to avoid cross-contamination. The cowpats were protected from UV influences during transportation.

The field exposed cowpats were removed from the plastic saucers and placed on grassland plots with no previous history of livestock grazing activity at the University of Stirling. Cowpats were set out 1 m apart, with each seasonal experiment occupying an adjacent row to avoid placement of fresh faeces on previously contaminated grassland. The placement of the cowpats was such that it prevented any wash-off of *E. coli* from faeces being able to contaminate another cowpat should heavy rainfall occur. The field exposed cowpats were subjected to seasonal outdoor temperature and rainfall conditions. The 30-year long term annual average values for rainfall, maximum temperature and minimum temperature are 1019 mm, 12.9 °C and 5.6 °C, respectively^38^.

By contrast, the polytunnel protected cowpats experienced zero rewetting from rainfall and warmer ambient air temperatures, and for practicality these cowpats remained on the plastic saucers throughout the experiment. The polytunnel was constructed of 20 Gauge (180 micron) UV stabilised Visqueen Politherm Plus AF polythene. This incorporates infra-red properties for retaining heat and anti-fog characteristics to prevent fogging/dripping. In summer, therefore, the polytunnel environment mimicked a drought and warming treatment while during winter it represented a more mild winter treatment than that experienced in the field. Each cowpat was repeatedly sampled throughout each of the seasonal sampling programmes, with higher frequency sampling in the immediate period following excretion. This resulted in samples being collected on day 0, 0.5, 1, 3, 4, 7, 8, 10 and 14 before a lower frequency of sampling was adopted. Seasonal experiments lasted until either *E. coli* fell below limits of detection (100 CFU per g fresh (wet) weight faeces) or the faecal material was no longer intact, thus preventing sample acquisition. Therefore, experiments ranged from 66 to 98 days in duration. During each seasonal experiment at least 13 faecal subsamples were removed from each replicate cowpat to monitor the concentrations of *E. coli* over time. When conditions allowed (e.g. cowpats remained intact, or cell numbers remained high) up to 15 faecal subsamples were obtained for analysis.

On every sampling occasion approximately 15 g of faeces (3 × 5 g subsamples) was randomly sampled from each cowpat using a sterile spatula (70% IMS, rinsed with sterile water) and placed into sterile 50 mL centrifuge tubes. To avoid sampling the surface crust of the dairy faeces the samples were extracted from the core of the cowpats. Samples were assumed to be well mixed and homogeneous following faecal passage through the ruminant digestive system and gut^31^. All microbial analysis was initiated within one hour of samples being collected.

### Culturing of E. coli

Two grams of fresh faeces was used for microbial analysis and the remainder (~13 g) was used to determine the gravimetric water content by drying at 105 °C for 24 h (until constant mass) and weighing the residual. For culture-based analysis, one gram of faeces was transferred to 9 mL of sterile phosphate buffered saline (PBS, Fisher Bioreagents, New Jersey, USA) and then thoroughly mixed using an orbital shaker (160 rpm for 60 minutes at ambient temperature) to disperse cells from the faecal matrix. Further serial 1:10 dilutions were then made as appropriate to ensure capture of between 20 to 200 colony forming units (CFU) once the sample had been transferred to an agar growth medium. To get to this stage, 1 mL of each serially-diluted sample was washed through a filtration unit (Sartorius, Germany) with ~20 mL of sterile PBS. Cellulose acetate membrane filters of 0.45 micron pore size (Sartorius Stedim Biotech., Goettingen, Germany) were aseptically transferred to Membrane Lactose Glucuronide Agar (MLGA) (CM1031, Oxoid, Basingstoke, UK) and incubated inverted at 37 °C (±0.2 °C) for 18–24 h for the determination of presumptive *E. coli*. Equipment was flame sterilised between samples and method blanks (i.e. sterile PBS) used to confirm aseptic technique and the sterilisation procedure between samples. The limit of detection was 100 CFU per g fresh weight faeces. All sample analysis was performed in duplicate.

### Statistical analysis

All *E. coli* counts underwent log_10_ transformation prior to statistical analysis but not all transformed distributions of *E. coli* were normally distributed as determined using the Kolmogorov-Smirnov goodness of fit test. Non-parametric tests (Mann-Whitney and Kruskal Wallis) were therefore used to test for differences between treatments. Differences at the P < 0.05 level (95% confidence interval) were considered statistically significant. Linear regression was used to investigate relationships between the rate of change of *E. coli* populations and the internal temperature of the faecal environment in the first three days post defecation (where the assumption of normal distribution was satisfied) (Minitab 16.0 software, Minitab Inc., PA, USA).

## Additional Information

**How to cite this article**: Oliver, D. M. and Page, T. Effects of seasonal meteorological variables on *E. coli* persistence in livestock faeces and implications for environmental and human health. *Sci. Rep.* **6**, 37101; doi: 10.1038/srep37101 (2016).

**Publisher’s note:** Springer Nature remains neutral with regard to jurisdictional claims in published maps and institutional affiliations.

## Figures and Tables

**Table 1:** Meteorological characteristics associated with the seasonal experiments (rainfall and air temperature data sourced from local weather station situated on University of Stirling campus).

	Total rainfall (mm)	Air temp (°C)			Polytunnel temp (°C)			Field cowpat temp (°C)		Polytunnel cowpat temp (°C)	
		Mean	Min	Max	Mean	Min	Max	Min	Max	Min	Max
2012											
Spring	174.4	8.9	−2.8	25.8	14.6	−3.0	56.5	*	*	2.0	43.0
Summer	435.6	13.8	4.6	23.2	20.3	5.5	54.0	6.6	38.9	10.0	46.5
Autumn	159.0	9.5	−1.1	20.6	11.2	−2.5	42.0	−0.5	25.0	0.5	27.5
Winter	241.8	4.6	−2.4	12.3	3.3	−6.5	16.0	−4.0	9.0	−1.0	10.5
2013											
Spring	114.2	5.8	−4.0	21.2	10.0	−6.0	50.0	−0.5	24.5	−0.5	37.5
Summer	95.7	16.3	7.7	30.1	23.3	7.5	56.0	6.0	39.5	11.5	50.5
Autumn	257.6	10.5	−3.6	22.5	14.0	1.0	46.5	4.0	24.5	4.0	26.5
Winter	312.8	5.7	−0.9	12.8	5.0	−3.5	15.5	−0.5	12.5	0.5	13.0

**Table 2:** Characteristics of observed *E. coli* growth phases recorded during the first period of post defecation growth.

Treatment	Season	Year	Mean peak *E. coli* during growth phase (log_10_ CFU g^−1^ dw faeces)	Mean increase in concentration from excreted *E. coli* concentration (log_10_ CFU g^−1^ dw faeces)	Days taken to reach peak *E. coli* concentration of growth (d)
Field	Summer	2012	7.90	1.01	16
Polytunnel	Spring	2012	7.90	0.92	49
Polytunnel	Summer	2012	7.65	0.96	10
Polytunnel	Autumn	2012	8.24	1.18	8
Field	Summer	2013	8.50	1.19	8
Polytunnel	Spring	2013	7.40	0.92	49
Polytunnel	Summer	2013	8.55	1.13	7
Polytunnel	Autumn	2013	7.37	0.91	7

Peak *E. coli* = highest value of *E. coli* that was recorded subsequent to values determined upon excretion.
